# Establishing an *in vivo* large animal model of one-lung ventilation and operative lung trauma

**DOI:** 10.1371/journal.pone.0335012

**Published:** 2025-10-27

**Authors:** Catherine Giffin, Jay Kormish, Martha Hinton, Shyamala Dakshinamurti, Ruth Graham, Biniam Kidane

**Affiliations:** 1 Max Rady College of Medicine, University of Manitoba, Winnipeg, Manitoba, Canada; 2 Department of Surgery, Rady Faculty of Health Sciences, Max Rady College of Medicine, University of Manitoba, Winnipeg, Manitoba, Canada; 3 Children’s Hospital Research Institute of Manitoba, Winnipeg, Manitoba, Canada; 4 Department of Physiology and Pathophysiology, Rady Faculty of Health Sciences, Max Rady College of Medicine, University of Manitoba, Winnipeg, Manitoba, Canada; 5 Department of Anesthesiology, Perioperative and Pain Medicine, Max Rady College of Medicine, University of Manitoba, Winnipeg, Manitoba, Canada; Faculty of Medicine Universitas Indonesia - CIPTO Mangunkusumo General Hospital, INDONESIA

## Abstract

**Background:**

Respiratory complications, including acute lung injury (ALI) and acute respiratory distress syndrome (ARDS), are important causes of morbidity and mortality among lung surgery patients. Lung surgery introduces surgical and atelectatic trauma to the operated lung, while one-lung ventilation (OLV) applied to the contralateral lung is also a suspected mechanism of ventilator-induced lung injury (VILI). Our goal was to develop a large animal model to assess the relative lung injury induced by surgical and ventilator trauma during left upper lobectomy in juvenile pigs.

**Methods:**

Sixteen pigs (24–32 kg) were randomly assigned to one of three OLV exposure groups. The control group (n = 5) was exposed to lung-protective ventilation (LPV) during OLV, the second group (n = 5) was exposed to potentially injurious ventilation (IMV) during OLV using higher tidal volume and peak airway pressure and the third group (n = 6) was exposed to hyperoxia with protective ventilation (LPV-HO) for the duration of OLV and surgery.

**Findings:**

We describe the surgical and ventilation methods for a successful lung surgery pilot for a porcine OLV model. Initial surgeries show that our protocol is effective in reproducibly maintaining peak airway pressures, tidal volumes and oxygen delivery according to the parameters of LPV, IMV and hyperoxia during OLV. Bronchoalveolar lavage fluid IL-6 was elevated in response to IMV during OLV, hyperoxia and surgical exposure.

**Conclusions:**

We describe a reproducible protocol for an *in vivo* large animal model of OLV lung surgery with a protective and two injurious mechanical ventilation arms with collection of physiologic data and biospecimens.

## 1 Introduction

Respiratory failure attributed to acute lung injury (ALI) and acute respiratory distress syndrome (ARDS) is a significant cause of postoperative mortality among patients undergoing lung surgery. Although varying depending upon patient population, ALI or ARDS occurs in 0.6-13.8% of adult lung surgery patients and is associated with 72.5% of postoperative deaths [1–11]. ARDS and ALI, now defined as mild ARDS, are complex inflammatory pathologies of the respiratory system defined by acute onset, hypoxemia that is refractory to oxygen therapy, diffuse infiltrates detected by chest radiograph and respiratory failure not caused by cardiac failure or fluid overload [12]. Inflammation and tissue injury are important mediators for the development of ALI and ARDS and it is suspected that ventilation-induced lung injury (VILI) and surgical trauma contribute to the occurrence and severity of these pathologies through proinflammatory mechanisms [2,13–16]. Mechanical ventilation alone contributes to lung injury and inflammation through multiple physical insults including volutrauma, barotrauma, and atelectotrauma [17–24]. Biotrauma is suspected to be caused by immune cell recruitment and oxidative stress from an increased fraction of inspired oxygen, *i.e*., a FiO2 > 21%, although the associated clinical outcomes remain controversial [25–32]. Current models incorporate multiple organ insults that converge to activate proinflammatory profiles further increasing the risk of ALI and ARDS due to VILI [16,33]. Mechanical ventilation is particularly injurious in the setting of lung resection surgery, when one-lung ventilation (OLV) is required as all the mechanical forces are delivered to only one lung [34,35]. The risk of volutrauma is increased as the entire tidal volumes (VT) is applied to a single dependent lung. The risk of atelectotrauma increases with the increased respiratory rate required to maintain a constant minute ventilation (Ve) when VT is decreased during the two-lung to one-lung ventilation transition. The operated lung is subject to surgical trauma and deflation. The latter may cause an additional level of injury due to hypoxic pulmonary vasoconstriction (HPV)-driven hypoxia and ischemia-reperfusion injury following reinflation [34,36].

The frequency of respiratory complications after lung resection surgery is associated with patient comorbidities such as advanced age, male sex, poor preoperative respiratory function, and extremes in BMI, and perioperative factors including surgery duration and the location and extent of lung resection [3,8,9,37–39]. As such, current human clinical models are limited by the heterogeneity of the lung surgery patient population, the ethical need to avoid potentially harmful mechanical ventilation conditions intraoperatively, and the inability to conduct histologic assessment of the ventilated lung. This has led to difficulties in delineating specific deleterious aspects of OLV and as a consequence, difficulties in designing true evidence-based lung-protective ventilation strategies [40–43]. Establishment of an animal model allows for careful control of ventilator settings and FiO2 to determine the independent contribution of each factor and provides the opportunity to directly observe inflammatory changes in biological specimens and histological samples from both lungs. In this study, we aimed to establish a preclinical *in vivo* experimental pig model that resembles a clinical human model of one-lung ventilation and lung resection.

A pig model was chosen as it represents a good large animal model for lung surgery from an anatomic and physiologic perspective [44–46]. It is an accepted model for assessing ALI and VILI [47–50], with significant local experience for non-surgical ALI studies [51–53]. Pig lung anatomy is a reasonable approximation of human anatomy (*i.e.,* in size and structure) and is used for training surgeons in thoracic surgical techniques. Because of similar anatomy and physiology, the surgical trauma exerted during lobectomy is also analogous to the human lung procedure.

## 2 Methods

### 2.1 Experimental design

**Fig 1:**
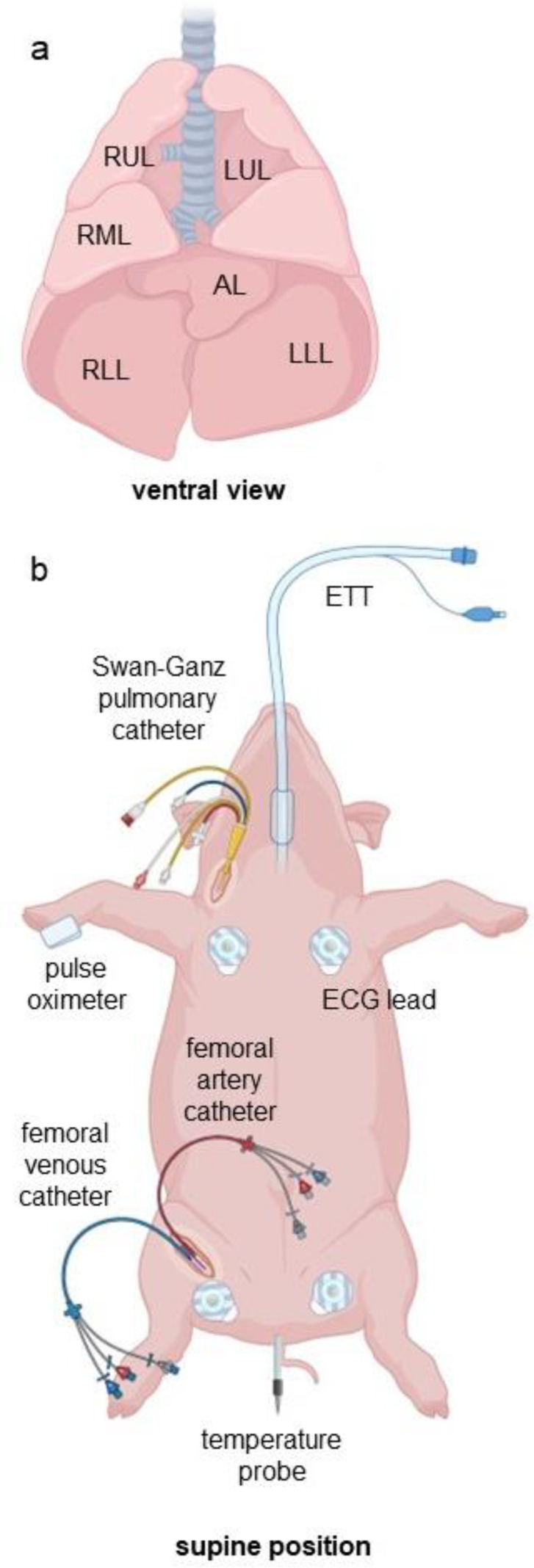
Schematics of porcine lung anatomy and animal preparation for lung surgery. (a) Ventral view of porcine lung anatomy. Left upper lobe (LUL), left lower lobe (LLL), right upper lobe (RUL), right middle lobe (RML), right lower lobe (RLL) and the accessory lobe (AL) are indicated. Lobular and bronchial branching structure of the pig is similar to humans except for the AL lobe that is only present in the pig anatomy and the RUL bronchus which branches off early and separately in all pigs but has a variable placement in some humans. (b) Schematic of animal preparation prior to the start of lung surgery and the initiation of OLV. Probe, catheter and sensor placement are similar to those used by the surgical team.

Twenty-four three-month-old farm-bred pigs weighing on average 27 kg (range 24–32 kg) were procured from an approved farm by the University of Manitoba Animal Services and randomly assigned to either the control group with lung protective ventilation and low inspired oxygen (FiO2) (LPV; n = 8), an injurious mechanical ventilation group (IMV; n = 9) with low inspired oxygen (FiO2), or the lung-protective ventilation and hyperoxia group (LPV-HO; n = 7). Formal sample size calculations were not performed as there is no data for us to have made reliable sample size calculations. We designed this as a pilot study to be able to ensure that we have a reliable large animal model of OLV injury during lung surgery and then to generate data so that we can inform sample size calculations for future larger studies. This is a feasibility-oriented exploratory study. The sex of the pigs was randomized, and all pigs underwent left upper lobectomy (Fig 1a). Three pigs died before the experimental endpoint. Two pigs with a pre-existing lung infection and three pigs that did not meet the experimental thresholds for LPV or IMV were removed from analysis. Following these exclusions, 16 pigs were included in the final analysis for LPV n = 5, IMV n = 5 and LPV-HO n = 6. Lung protective ventilation during OLV was defined as a VT of 6 mL/kg to provide a peak inspiratory pressure (PIP) <30 cm H2O, a positive-end expiratory pressure (PEEP) of 5 cm H2O, an inspiratory: expiratory (I:E) ratio of 1:2 and FiO2 < 50%. The IMV group was ventilated with an increased VT of 10-12mL/kg to achieve a PIP ≥ 30 cmH2O. PEEP = 5 cm H2O and FiO2 < 50% were maintained at levels similar to LPV group. The LPV-HO group was ventilated with identical parameters as the LPV group but with FiO2 increased to 100%. Variable ventilation exposures were limited to the OLV period of the procedure.

### 2.2 Ethical considerations

This study was approved by the Bannatyne Campus Animal Care Committee [Protocol Reference Number: 21−013 (AC11692)]. This study was not a survival experiment. It was an intraoperative experiment lasting a maximum of three hours, where all animals (pigs; *Sus domesticus*) were totally monitored throughout the procedure, including all behaviour parameters. All animal welfare considerations were taken into consideration to minimize suffering and distress, and animals were under anesthesia throughout the entire time. At the end of the procedure, pigs were euthanized with 100 mEq/kg of potassium chloride (KCl) injected directly into the heart, ensuring immediate cardiac arrest and painless death. All pigs died during the procedure. 3 pigs died at the beginning of the experiment while under anesthesia (1 because of severe pneumonia causing respiratory failure under anesthesia; 2 because of great vessel injury causing immediate exsanguination). Importantly, all research personnel directly involved in the procedure were appropriately trained in animal care and handling procedures in accordance with the University of Manitoba’s animal care and handling guidelines.

## 3 Procedure

### 3.1 Animal preparation and two-lung ventilation

3.1.1 For each experiment the pig was weighed and sedated with intramuscular xylazine (1 mg/kg), ketamine (10 mg/kg) and atropine (0.01 mg/kg). Anesthesia was induced with inhaled isoflurane (4%) by nose cone. Under direct laryngoscopy, 1% xylocaine spray was applied to the vocal cords to prevent laryngospasm and a 7.0 cuffed endotracheal tube (ETT) was inserted.

3.1.2 After intubation, total intravenous anesthesia (TIVA) was initiated using an infusion of propofol, and ketamine (10–15/2.5-5 mg/kg/hr) titrated to maintain stable hemodynamics and lack of response to surgical stimulation. TIVA permitted the use of an intensive care ventilator (Esprit®) with the capability of measuring respiratory mechanics. Lack of respiratory effort during mechanic measurements was ensured with intermittent rocuronium 1 mg/kg intravenous (IV) for muscle paralysis.

3.1.3 The animal was placed on an underbody warming air blanket (Bair Hugger®) to maintain body temperature.

3.1.4 Two-lung ventilation was maintained with tidal volumes (VT) of 10 ± 2 ml/kg, inspiration/expiration (I:E) ratio of 1:2, peak inspiratory pressure (PIP) 20 ± 5 cm H2O, positive end-expiratory pressure (PEEP) of 5 ± 0.5 cmH2O, a fraction of inspired oxygen (FiO2) of 30–50% and the respiratory rate adjusted to maintain a normal PaCO2 (*i.e.*, 40 ± 5 mmHg).

### 3.2 Monitoring

3.2.1 Electrocardiogram (ECG) leads, a peripheral oxygen saturation monitor, and a temperature probe were placed for continuous monitoring (Fig 1b).

3.2.2 A femoral arterial catheter and femoral venous catheter were placed *via* cut-down for continuous measurement of arterial blood pressure and blood gas sampling (Fig 1b). The cut-down site was infiltrated with Bupivacaine (0.25%) for local anesthesia. A 5.0 Swan Ganz thermodilution pulmonary artery catheter (Fig 1b) was placed *via* cut-down in the right internal jugular vein for continuous monitoring of pulmonary artery pressures and intermittent blood gas sampling. The cut-down site was infiltrated with Bupivacaine (0.25%) for local anesthesia.

3.2.3 The animal was allowed to stabilize under TLV for 15 minutes and the TLV baseline hemodynamics, ventilator settings and respiratory mechanics were recorded for the first procedure timepoint. Arterial and venous blood samples were obtained for blood gas analysis and plasma separation. A fiberoptic bronchoscope was wedged into the right and left mainstem bronchus sequentially and bronchoalveolar lavage fluid (BALF) samples were obtained from each lung by instillation of 10–15 ml sterile normal saline followed by aspiration. Samples were stored on ice for later analysis.

### 3.3 One-lung ventilation protocols

3.3.1 With the animal in the supine position, a bronchial blocker (EZ Blocker) was placed under bronchoscopic guidance and the left-sided balloon was inflated to initiate OLV.

3.3.2 The OLV protocol was initiated according to assigned exposure group as outlined below:

i. **Lung-protective ventilation and low FiO2 control group (LPV)**: OLV tidal volume (VT) was adjusted to 6 ml/kg. Baseline minute ventilation (Ve) was maintained by increasing the respiratory rate. An I:E ratio of 1:2, PIP < 30 cmH2O, PEEP of 5 ± 0.5 cmH2O and FiO2 < 50% were maintained throughout the OLV procedure.
ii. **Injurious ventilation and low FiO2 group (IMV):** VT was adjusted to 10–12 ml/kg to provide a PIP > 30 cmH2O. An I:E ratio of 1:2, PEEP of 5 ± 0.5 cmH2O and FiO2 < 50% were maintained similar to the LPV group above.
iii. **Lung-protective ventilation and hyperoxia group (LPV-HO):** OLV VT was adjusted to 6 ml/kg. Similar to the LPV group, baseline minute ventilation (Ve) was maintained with an increase in respiratory rate. An I:E ratio of 1:2, PEEP of 5 ± 0.5 cmH2O and FiO2 = 100% were maintained throughout the OLV procedure.

3.3.3 Animals were allowed to stabilize under OLV for 15 minutes and the OLV supine series of hemodynamics, ventilator settings and respiratory mechanics were recorded for the second experimental timepoint. Arterial and venous blood samples were obtained for blood gas analysis.

3.3.4 The animal was placed in the right lateral decubitus position and allowed to stabilize under OLV for 15 minutes. The OLV lateral series of hemodynamic and ventilator readings were recorded for the third experimental timepoint. Arterial and venous blood samples were obtained for blood gas analysis.

### 3.4 Surgery

**Fig 2:**
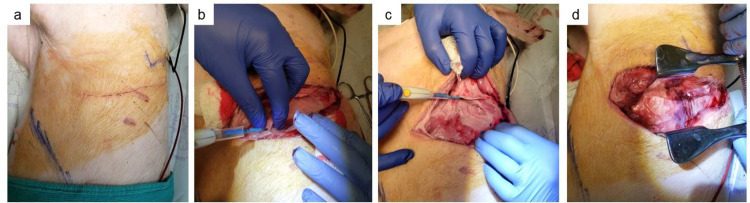
Left posterolateral thoracotomy. (a) Incision marking 6th intercostal space (b) dissection of the subcutaneous and muscular tissues. (c) Dissection into intercostal space. (d) Placement of Finochietto retractor to spread rib spaces.

3.4.1 A left lateral thoracotomy (Fig 2) was performed in standard fashion.

3.4.2 The ribs were palpated to landmark and enter the 6th intercostal space (Fig 2a).

3.4.3 The initial incision was performed using electrocautery with a constant waveform denoted commonly as “cutting” mode (Fig 2b).

3.4.4 The subcutaneous tissue was dissected using the same technique. The cautery was placed at the superior aspect of the inferior rib to avoid damage to the neurovascular bundle (Fig 2c).

3.4.5 An open metal rib spreader (Finochietto retractor) was placed within the incision to secure a clear view of the left upper lobe of the lung.

**Fig 3:**
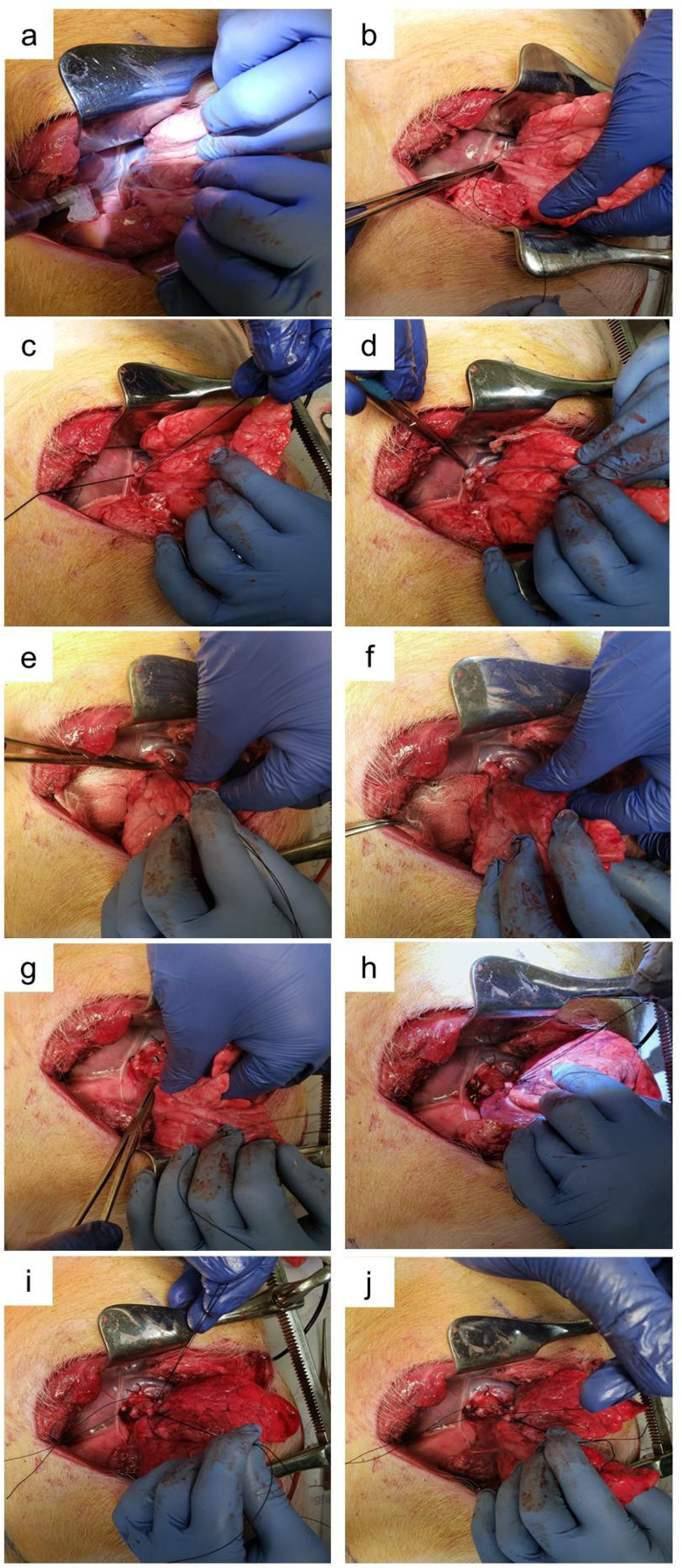
Isolation of left upper lobe. (a) Left phrenic nerve block. (b) Isolation of superior pulmonary vein, (c) ligation and (d) transection of superior pulmonary vein. e) to f) Isolation, ligation and transection of pulmonary artery branches. (g) to (h) Isolation, ligation and transection of left upper lobe bronchus. (i) and (j) Isolation and ligation of lingular pulmonary artery branch.

3.4.6 Using bupivacaine (0.25%), an intercostal and left phrenic nerve block was performed prior to proceeding to anatomical resection of the left upper lobe (Fig 3a). Total bupivacaine dose was limited to 2.5 mg/kg divided between all infiltration sites.

3.4.7 The superior pulmonary vein was isolated, ligated and transected (Fig 3b–d).

3.4.8 Pulmonary artery isolation, ligation and transection was performed sequentially, starting with the anterior truncus branch and ending with the lingular branches (Figs 3e, f, and 3i, j).

3.4.9 The left upper lobe bronchus was isolated, ligated and transected (Fig 3g, h).

**Fig 4:**
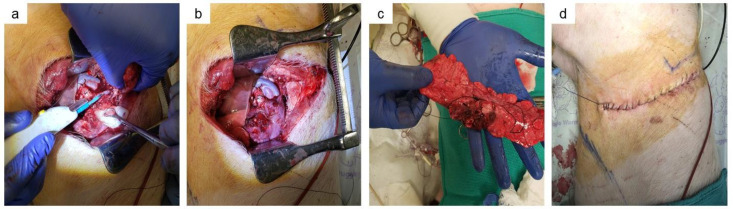
Completion of surgery. (a) Cautery for completion of parenchymal fissure. (b) post lobectomy space. (c) Removing lung specimen. (d) wound closure after cessation of OLV and re-inflating left lower lobe for TLV.

3.4.10 The parenchymal fissure was dissected with electrocautery. The left upper lobe was removed from the thoracic cavity and placed in Krebs-Henseleit buffer (containing in mM: 112.6 NaCl, 25 NaHCO3, 1.38 NaH2PO4, 4.7 KCl, 2.46 MgSO4, 7 H2O, 5.56 dextrose; pH 7.4) (Fig 4) one ice for postoperative analysis.

3.4.11 Prior to cessation of OLV, the OLV intraoperative series of hemodynamics and ventilator settings were recorded for the fourth timepoint. Arterial and venous blood samples were obtained for blood gas analysis.

3.4.12 Wound closure was completed with a running suture (Fig 4d).

3.4.13 To terminate OLV ventilation, the endotracheal blocker was removed. Two-lung ventilation was resumed.

3.4.14 The animal was allowed to stabilize under TLV for 15 minutes and the final and fifth timepoint was recorded including the TLV postoperative hemodynamics, ventilator settings and respiratory mechanics. Arterial and venous blood samples were obtained for blood gas analysis and plasma separation. A second BALF sample was obtained from both the operated and ventilated lung as previously described.

### 3.5 Termination of experiment and lung explant

**Fig 5:**
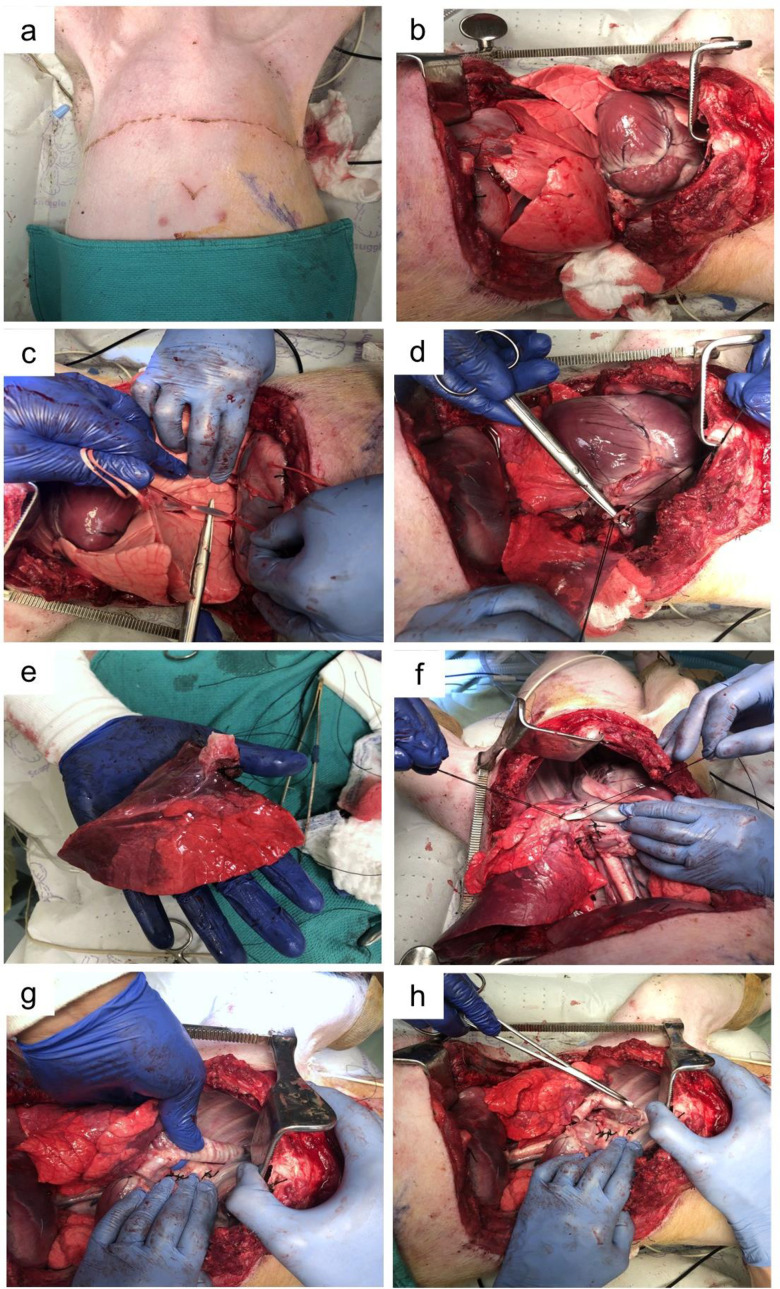
Removal of lungs en bloc via bilateral clamshell thoracotomy. (a) Bilateral clamshell thoracotomy marking. (b) Bilateral clamshell thoracotomy incision. c) Isolation, ligation and transection of left lower lobe veins and arteries. (d) Isolation, ligation and transection of inferior vena cava. (e) Transection of left mainstem bronchus and extraction of left lower lobe. (f) Isolation, ligation and transection of right lung veins and arteries. (g) Isolation of trachea proximal to right upper lobe take-off. (h) Explant of trachea and right lung.

3.5.1 A bilateral clamshell thoracotomy was performed (Fig 5).

3.5.2 KCl (100 mEq/kg) was injected directly into the heart and was observed to ensure cardiac standstill.

3.5.3 The inferior vena cava was ligated (Fig 5d).

3.5.4 Ventilation was terminated and all of the right lung and remaining left lower lobe were removed *en bloc* (Fig 5) with minimal handling and stored in Krebs-Henseleit buffer on ice for postoperative analysis.

## 4 Data collection and analysis

4.1 Procedure timepoints were recorded including preoperative sedation, intubation, the start and termination of OLV and the postoperative two-lung ventilation end point. Hemodynamic and ventilatory parameters were recorded at five time points: baseline (TLV), OLV (supine), OLV (LLD position), OLV (post lung resection), post-surgical (TLV).

4.2 Parameters included heart rate, mean arterial blood pressure, central venous pressure, pulmonary arterial pressure, body temperature, end-tidal CO2 (ETCO2), O2 saturation (SpO2), fraction of inspired oxygen (FiO2), tidal volume (VT), respiratory rate (RR), minute ventilation (Ve), inspiratory: expiratory ratio (I:E), peak inspiratory pressure (PIP), positive end-expiratory pressure (PEEP), mean airway pressure (MAP), static compliance and resistance as determined by the average of three separate measurements using the single breath technique.

4.3 Blood gas analyses performed included arterial and mixed venous pH, arterial and venous partial pressure of O2 (PaO2) (PvO2), arterial and venous partial pressure of CO2 (PaCO2) (PvCO2), and arterial and venous bicarbonate concentration (cHCO3-). Hemodynamic and ventilation data were analyzed using one-way and two-way ANOVA repeated measures (RM) or mixed model analysis with significant P-values indicated for one-way group (p1) and time (p2) and two-way group: time (p3) interactions. An uncorrected Fishers Least Significant Difference (LSD) was applied for the five individual time point comparisons between OLV groups. Significance was accepted at the p < 0.05 level.

4.4 Pre and post-OLV serum and BALF samples were analyzed for cytokine abundance through the multiplex Luminex xMAP technology and Luminex 200 system for five pigs from each OLV group (Fig 8).

**Fig 6:**
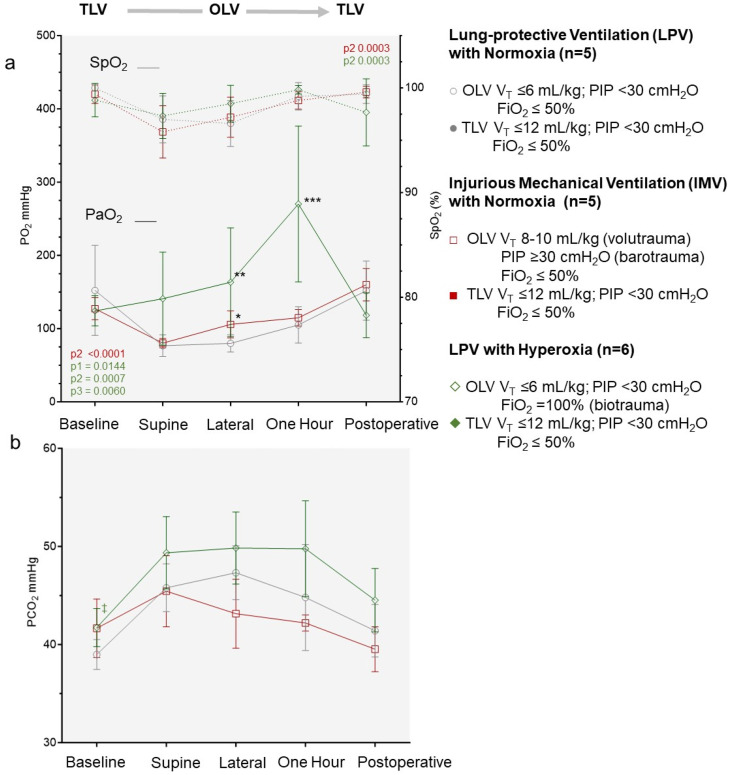
Lung protective and injurious mechanical ventilation outcomes. Ventilatory parameters applied in the lung-protective ventilation (LPV), injurious mechanical ventilation (IMV) and LPV with hyperoxia (LPV-HO) exposure groups including mean (a) tidal volume (VT) (b) peak inspiratory pressure (PIP) (c) respiratory rate (RR) (d) mean airway pressure (MAP) and (e) minute ventilation (Ve) delivered during lung surgery within the three exposure groups. Thresholds predicted for injurious volutrauma and barotrauma are indicated in a and b respectively. Significant P values include ANOVA RM analysis for group type (p1), OLV procedure time (p2) and group type: procedure time (p3). Significant P values from a from a Fishers LSD group comparison across all procedure time points are indicated as * < 0.0001, ** 0.0002 *** 0.0001, † 0.0012, †† 0.0006, ††† 0.0127, †††† 0.0498 # 0.0091, ## 0.0382 and ### 0.0422.

**Fig 7:**
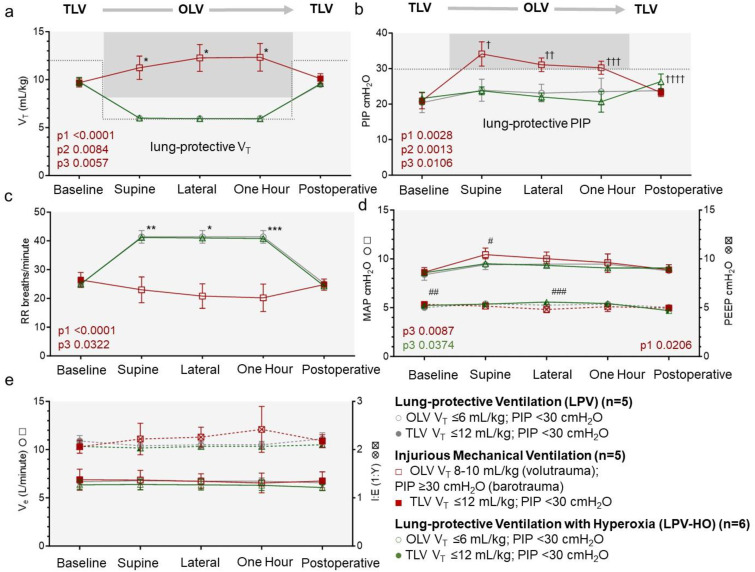
Blood gas analysis in animals subjected to lung protective and injurious mechanical ventilation. Arterial and venous blood gas analysis comparing partial pressures for (a) O2 and (b) CO2 for the three exposure groups. Percent oxygen saturation (SpO2) recorded by a pulse oximeter is compared to blood gas analysis in (a). Significant P values include ANOVA RM or Mixed model analysis for group type (p1), OLV procedure time (p2) and group type: procedure time (p3). Significant P values from a from a Fishers LSD group comparison (LPV to IMV or LPV-HO) across all comparison procedure time points are indicated as * 0.0342 **0.0395 *** 0.0114 and ‡ 0.0274.

**Fig 8:**
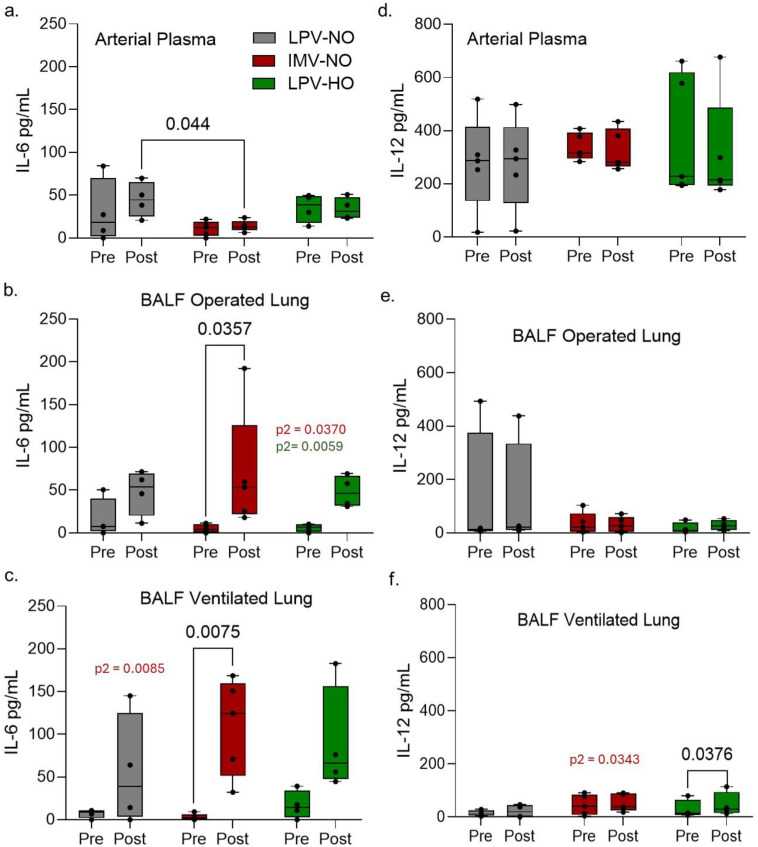
IL-6 and IL-12 cytokine analysis within the plasma and BALF samples. IL-6 and IL-12 concentration was quantified for the pre-OLV and post-OLV sampling time points in the arterial plasma (a,d), and BALFs sampled from the operated (b,e) and ventilated (c,f) lungs. Significant P values include ANOVA RM for group type (p1), OLV procedure time (p2) and group type: procedure time (p3). Significant P values from a from a Fishers LSD group comparison (LPV to IMV or LPV-HO) across all comparison procedure time points are indicated. Box and whisker plots indicate the 25-75% percentile range with minimum and maximum values.

4.5 Pre- and post OLV cytokine concentrations were analyzed using one-way and two-way ANOVA repeated measures (RM) with significant P-values indicated for one-way group (p1) and time (p2) and two-way group: time (p3) interactions. An uncorrected Fishers Least Significant Difference (LSD) was applied for individual time point comparisons between OLV groups.

## 5 Results

Maintaining an open lung while minimizing tidal volume and airway pressure excursions forms the basis of a lung-protective mechanical ventilation strategy. In the LPV group in the current study, mean VT, PIP and PEEP were maintained at 6.0 (range 5.6-6.4) mL/kg, 23.5 (range 20.2-29.7) cmH2O and 5.3 (range 4.9-5.5) cmH2O respectively, during OLV (Fig 6a, b). During the TLV to OLV transition, an increase in RR from 25 to 41 (range 40–45) breaths/minute was required to maintain a baseline mean minute ventilation (Ve) of 6.7 (range 6.3-7.1) L/minute. Whether even lower VT or higher PEEP levels may be advantageous, or more “protective” during OLV could be tested in future studies.

The LPV-HO group had identical ventilator settings as the LPV group but with 100% FiO2 delivered during the period of OLV. OLV exposures in the IMV group included potential volutrauma (VT 10–12 mL/kg) and barotrauma (PIP > 30 cmH2O). In the IMV group, mean VT was increased to 11.9 (range of 9.4-14.3) mL/kg resulting in an increase in mean PIP to 31.8 (range 27.2-38.0) cmH20. The baseline minute ventilation of 6.7 (5.1-7.7) L/minute in this group was maintained by a reduction in the mean RR to 21 (range 15–30) breaths/minute at the transition point. The inspiratory: expiratory (I:E) ratio and MAP did not differ significantly between the LPV and IMV groups with the exception of the MAP supine position OLV timepoint (Fig 6d). Positive-end expiratory pressures (PEEP) were marginally increased at the first OLV time point in the IMV group (Fig 6d).

Peripheral oxygen saturation (SaO2) did not differ significantly between the LPV, IMV and LPV-HO groups (Fig 7a). After a slight fall in SaO2 with the initiation of OLV, all groups showed similar increasing SaO2 trends during OLV (p2 = 0.003). Arterial blood gas analysis confirmed higher PaO2 in the LPV-HO group (p1 = 0.0144) compared to the LPV control, and values increased during the OLV period (p2 = 0.007) (Fig 7a). Significant differences in PaO2 between the LPV and LPV-HO groups were not achieved until the second OLV sampling time point; this indicates that the hyper-oxygenation exposure was applied correctly during OLV No significant differences in PaCO2 were observed between the LPV, IMV and LPV-HO groups during the OLV procedure (Fig 7b). Comparison of OLV and operative time between groups showed no significant differences in exposure durations of OLV and operative time between groups.

Pre vs post OLV serum IL-6 and IL-12 concentrations were not significantly different with any OLV mode (Fig 8a and d) although IL-6 concentrations were significantly lower (p = 0.0440) in the IMV group post-OLV (n = 5) group compared to the LPV (n = 4) group (Fig 8a). Whether this small difference is of any clinical relevance is unclear. In contrast, BALF IL-6 concentration increased significantly in both the operated (p2 = 0.037) and ventilated lungs (p2 = 0.0085) in the IMV group compared to the LPV group (Fig 8b, c) A similar trend was observed in the LPV-HO group for both operated lung (p2 = 0.0059) and ventilated lung (p = 0.0511). An increased variability in post OLV IL6 concentrations is evident. A small but significant increase in IL-12 concentration was only detected in in the IMV group in the ventilated lung (p2 = 0.0343) when compared to LPV group (Fig 8f). The small sample size in this pilot study and increased variability in post OLV cytokine concentrations may have limited the ability to demonstrate more robust differences in cytokine levels between LPV and IMV. An increase in IL-6 levels following lung surgery has been demonstrated to be a marker for increased risk for post-operative complications in human lung surgery patient population [2,16].

## 6 Discussion and conclusion

This pilot study demonstrates the ability to establish a successful large animal in vivo model for investigating lung injury during the complex interplay of lung surgery. Surgeries were completed successfully with good yield of physiologic and biospecimen data. Challenges exist in the exploration of lung inflammation in human models, and there is a need for a reliable animal model to test hypotheses and evaluate therapeutic modalities. This report outlines a reproducible protocol for a porcine model of one lung ventilation and lung surgery. To our knowledge, this is the first described and established model for this. Previously described models focused solely on one-lung ventilation without consideration of the impact of the lung surgery that necessitated the one-lung ventilation in the first place. In the only other study that was ever close to our model [47], Kozian et al utilized 30-minute stabilization period. However, their study did not assess operative surgical resection of the non-ventilated lung. In our study, the choice of 15-minutes was practical as it was based on the clinical and physiology expertise of a highly experienced cardiothoracic anesthesiologist with extensive experience in clinical OLV but also in pig ventilation experiments. Another practicality was that 15-minutes represented about 50% of the time required to do a surgical procedure. The use of the large animal also more closely approximates the human setting, which is novel.

In this study, proinflammatory responses, as manifested by the IL-6 and IL-12 responses documented, were evident in both the operated and ventilated lung with either increased ventilation pressures or increased FiO2 during OLV. As such, an injurious ventilation strategy, hyperoxia and surgical trauma may all contribute to the inflammatory response seen during lung surgery. We have demonstrated an ability to generate reliable data and which can serve as the basis for designing larger studies with more finely tuned targets and sample size calculations.

This study has limitations. The sample size is small and cannot be used to generate definitive conclusions and should be considered hypothesis-generating. Given that these are not transgenic animals, there will be heterogeneity in the pigs and thus it is possible that some of the differences identified in both clinical, physiological and molecular responses to the experiment may be heavily influenced by this baseline heterogeneity. To mitigate this, we chose 3-month old young pigs so as to limit the variability in the pulmonary (and other) exposures experienced by the pigs prior to surgery & OLV. This increases the homogeneity of the pig lungs and thus less confounding when trying to interpret the tissue damage that we observe after surgery and OLV. Additionally, future studies looking into ways to incorporate broader inflammatory and oxidative stress markers, such as investigating the cellular levels of reactive oxygen species would be highly beneficial in confirming our findings. This is something we plan to do in the next steps, but it is currently a limitation for this manuscript.”

This pilot study presents a stable and reproducible large animal *in vivo* model of OLV and lung surgery using distinct ventilation strategies. Target tidal volumes, inspiratory pressures and FiO2 were achieved, while maintaining good control of other physiologic and ventilatory variables. Surgeries were completed successfully with good yield of physiologic and biospecimen data. Challenges exist in the exploration of lung inflammation in human models, and there is a need for a reliable animal model to test hypotheses and evaluate therapeutic modalities. This report outlines a reproducible protocol for a porcine model of one lung ventilation and lung surgery. Use of this model could facilitate future studies to further define the most relevant parameters that contribute to post-operative lung injury and test potential lung protective strategies in a preclinical setting.

## 7 Institutional review board statement

The animal study protocol was approved by the Bannatyne Campus Animal Care Committee of The University of Manitoba (protocol code 21−013).
